# Effect of Post-Weld Heat Treatment on Microstructure and Hardness Evolution of the Martensitic Hardfacing Layers for Hot Forging Tools Repair

**DOI:** 10.3390/ma18174214

**Published:** 2025-09-08

**Authors:** Marzena Lachowicz, Marcin Kaszuba, Paweł Widomski, Paweł Sokołowski

**Affiliations:** Center for Materials Engineering and Metal Forming, Faculty of Mechanical Engineering, Wrocław University of Science and Technology, Lukasiewicza 5, 50-371 Wrocław, Poland

**Keywords:** post-weld heat treatment (PWHT), hardfacing, hot forging tools, martensitic microstructure

## Abstract

The study investigates the influence of post-weld heat treatment (PWHT) on the microstructure and hardness of hardfacing layers applied to hot forging tools. The research focuses on three tool steels (55NiCrMoV7, X37CrMoV5-1, and a modified X38CrMoV5-3) and uses robotized gas metal arc welding (GMAW) with DO015 filler material. It examines the structural and mechanical differences in the hardfaced layers before and after heat treatment involving quenching and tempering. The findings reveal that PWHT significantly improves microstructural homogeneity and hardness distribution, especially in the heat-affected zone (HAZ), mitigating the risk of crack initiation and tool failure. The study shows that untempered as-welded layers exhibit microstructural inhomogeneity and extreme hardness gradients, which negatively impact tool durability. PWHT leads to tempered martensite formation, grain refinement, and a more stable hardness profile across the joint. These improvements are critical for extending the service life of forging tools. The results underscore the importance of customizing PWHT parameters according to the specific material and application to optimize tool performance.

## 1. Introduction

The durability of tools used in industrial hot die forging processes is critical to the efficiency and cost-effectiveness of production. The relatively short service life of such tools leads to frequent downtime and replacement costs, negatively impacting both production output and the quality of forgings. Tool-related costs are estimated to account for 8–15% of total production costs, and for short series, even 30–40% [1]. Other analyses suggest that for complex and high-cost tools, this share may reach up to ~50% of total production costs [2]. Improving tool life is therefore directly linked to reducing unit costs and maintaining product quality and competitiveness.

During the forging process, the tools are subjected to extremely high loads [3]. Tool wear is directly related to destructive factors present during operation. The most common wear mechanisms include abrasive wear, thermo-mechanical fatigue, and plastic deformation [4,5,6]. As a result of these mechanisms, tools experience progressive wear, which reduces forging quality and raises production costs. To mitigate wear, various surface engineering techniques have long been used in the industry, such as thermo-chemical treatments (e.g., nitriding) or coatings applied via PVD/CVD methods [7,8,9,10,11,12,13,14]. Nonetheless, traditional and widely adopted gas nitriding continues to be the predominant method used in industrial practice [15,16,17,18,19]. However, there is growing interest in more advanced—albeit more expensive—methods that significantly extend tool life or allow for multiple reuse of the same tool [6]. One such approach is the refurbishment of forging tools to increase their operational lifespan. A common practice in this context is the mechanical reworking of tools by machining down the degraded surface layer. This method has its limitations, as it typically allows for only 2–3 refurbishment cycles. One of the most effective refurbishment strategies involves rebuilding worn areas using welding techniques [20].

Hardfacing involves applying a new metal layer to worn or damaged areas and partially melting the surface of the base material to form a strong metallurgical bond with the deposited layer [21,22,23]. The goal of such refurbishment is to achieve a layer with high resistance to wear and fatigue cracking, while minimizing the material input required to rebuild worn tool areas. It has been shown that hardfacing-based refurbishment can significantly extend die service life compared with traditional non-hardfaced tools. Literature reports even a several-fold increase in tool lifespan due to hardfacing—depending on the application, up to a threefold improvement has been observed [2,24]. Hardening also has a positive impact on other functional properties [25].

A range of advanced welding technologies is currently used for hardfacing-based refurbishment, including arc and laser methods. For example, automated MAG arc welding is widely applied, offering high material deposition rates. One of the most recent variants of this method is the cold metal transfer (CMT) process, characterized by significantly reduced heat input and stable metal droplet transfer, which results in a smaller heat-affected zone and lower dilution of the deposited layer in the base material compared to conventional arc welding methods [26]. Laser cladding technologies, such as powder-fed or wire-fed laser deposition, are also being continuously developed [27,28,29,30,31]. The laser process provides favorable conditions for forming the deposited layer—it ensures a low degree of dilution with the substrate and a high energy concentration, thereby limiting the heat-affected zone. Laser methods are especially suitable when high precision is needed—for instance, in the selective refurbishment of small, complex-shaped tools [32].

Hardfacing-based refurbishment of hot working tools is most often performed using filler materials with a chemical composition similar to the base material. These materials offer high resistance to wear and thermal fatigue, provided that the structure of the deposited layer is properly formed after the overlay welding process. Therefore, post-weld heat treatment (PWHT) is necessary to relieve residual welding stresses and ensure the desired structure of the deposited layer. The literature emphasizes that a single tempering cycle applied without prior hardening may not be sufficient to fully restore the desired properties—it is often recommended to apply a full hardening and tempering cycle, similar to tool steels, in order to obtain a favorable microstructure. The parameters of such heat treatment must take into account the composition of the deposited layer and the properties of the substrate material [33].

Forging tools operate under conditions of high mechanical and thermal loads; therefore, they must exhibit high hardness and impact toughness, as well as resistance to fatigue cracking. Currently, different research centers are actively working on the development of refurbishment technologies for forging tools and related applications. New filler materials are being introduced, including coatings reinforced with ceramic particles (e.g., hardfacing with TiC or WC dispersions to enhance wear resistance) [34,35,36]. Hardfacing leads to overheating of the substrate material and induces structural transformations in the heat-affected zone (HAZ). For this reason, significant research efforts are devoted to optimizing both hardfacing and post-weld heat treatment parameters. Heat treatment should ensure microstructural homogenization and eliminate unfavorable structural constituents that, under service conditions, exhibit insufficient resistance to dynamic loads. This aspect is critical for forging tools exposed to severe operating conditions. Since each tool differs in terms of material and working environment, the refurbishment process (encompassing both hardfacing and subsequent heat treatment) must be tailored individually.

It has been demonstrated that the development of a dedicated refurbishment technology for a specific tool requires an integrated selection of appropriate filler material, welding parameters, and heat treatment conditions in order to guarantee the required performance of the deposited layer. However, no well-defined criteria for parameter selection exist, highlighting the need for continued research in this area [33]. Such advancements would enable extension of tool service life, reduction of repair frequency and production downtime, thereby lowering overall manufacturing costs.

The proposed heat treatment corresponds to procedures traditionally applied to hot-work tool steels. However, it should be noted that hardfacing produces a material that is heterogeneous in both microstructure and chemical composition, which significantly differs from conventionally used tool materials. This necessitates the adjustment of heat treatment parameters to ensure appropriate microstructural and mechanical properties not only in the base material but also in the hardfaced layer, which became the subject of this investigation.

Conventionally, post-weld heat treatment (PWHT) is primarily aimed at reducing residual stresses generated during hardfacing that are the result of non-uniform heating and cooling. The accumulation of such stresses may lead to tool deformation, cracking, or reduced service life—effects that PWHT is intended to mitigate. In the present study, a comprehensive heat treatment procedure was proposed, specifically dedicated to tools after hardfacing, with the objective not only of reducing residual stresses but also of achieving a uniform microstructure and hardness across the entire tool cross-section.

## 2. Research Problem Statement

Post-weld heat treatment (PWHT) is recommended for many metal alloys, especially steels, as extensively discussed in the literature. For instance, ISO 15614 [37] specifies particular temperature ranges and holding times for different materials. These guidelines generally suggest single-step tempering at temperatures between approximately 300–900 °C. The numerous tools used in hot die forging processes, for which such PWHT procedures were applied in line with such recommendations, were studied. The analyses revealed significant issues, including cracking and the formation of unfavorable structures within deposited layers made from high-alloy hot-working tool steels [38,39].

**Figure 1:**
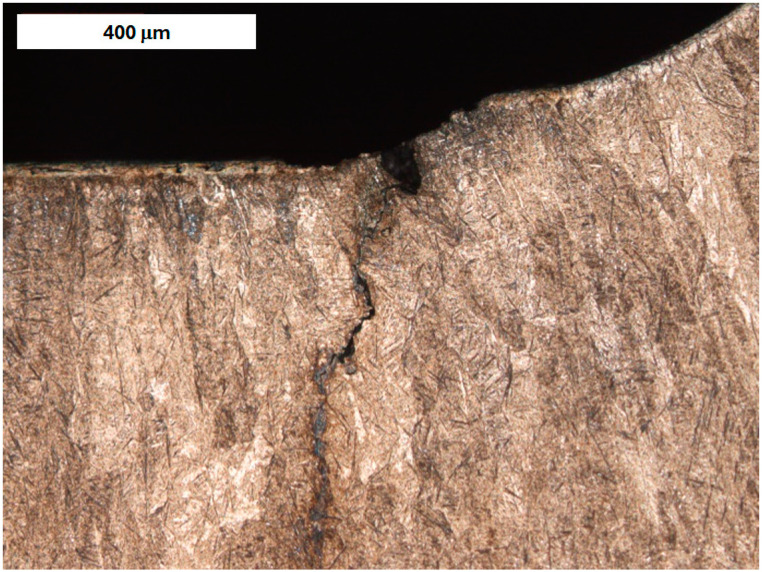
Surface crack in refurbished tool I (forging die for press hot forging). Light microscopy, etched condition.

**Figure 2:**
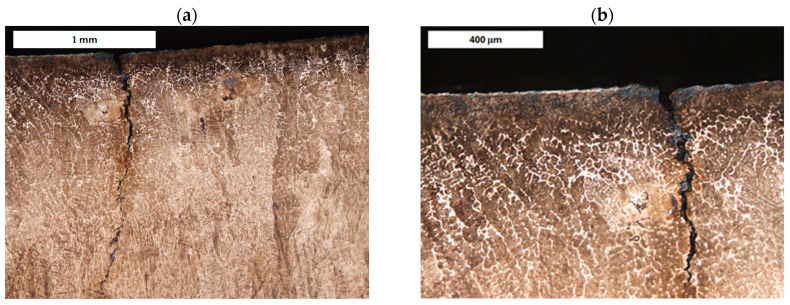
(**a**) Surface crack in refurbished tool II (forging die for press hot forging). (**b**) Enlarged detail of area from image a. Light microscopy, etched condition.

Cracks were observed to follow grain boundaries and appear in regions with increased retained austenite content (Figure 1 and Figure 2). These cracks are induced by thermo-mechanical fatigue. They originate near the surface and extend into the tool’s core. In addition to external factors like cyclic thermal and mechanical loads, another cause of these cracks is the excessively high hardness of the near-surface material, which sometimes exceeds 60 HRC [40].

**Figure 3:**
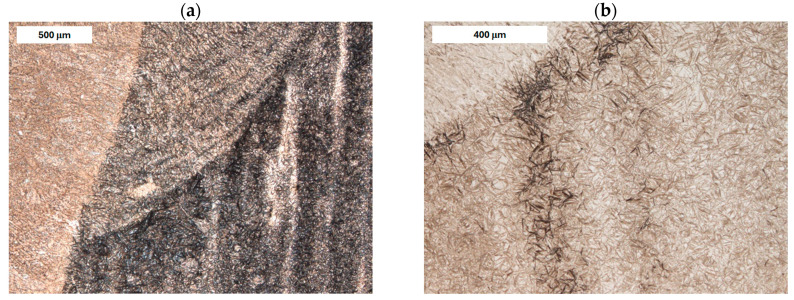
Microstructure of the deposit (**a**) and the HAZ (**b**) in the refurbished tool. Significant variation in material microstructure is visible. Light microscopy, etched condition.

Another major issue is the formation of non-martensitic structures, particularly as the significant cross-sections of tools promote slower cooling (see Figure 3). Microstructural inhomogeneity may result in varying mechanical strength and facilitate crack propagation.

## 3. Materials and Methods

This paper presents the results of hardfacing tests using the gas metal arc welding (GMAW) method, conducted on three grades of hot-work tool steels: 55NiCrMoV7, X37CrMoV5-1, and a modified X38CrMoV5-3 grade. The actual chemical composition of the base materials (BM) used in the study was determined using the GDOES method (Leco, St. Joseph, MI, USA, GDS 500A) and is provided in Table 1 [40]. All tested steels are chromium–molybdenum alloys with vanadium. The first two steels—55NiCrMoV7 (1.2714) and X37CrMoV5-1 (1.2343)—are standardized grades according to the ISO 4957:2018-09; Tool steels (Publisher: ISO 2018-06) standard. The third material is a non-standardized steel, whose composition is most similar to that of X38CrMoV5-3 (1.2367). However, in comparison to the tested steel, this grade contains slightly higher amounts of silicon and molybdenum. It also has a higher carbon content compared with the standard X38CrMoV5-3 grade. This allows it to form hard carbides, providing high abrasion resistance and thermal stability. 55NiCrMoV7 steel has a high nickel content, which provides excellent ductility. This should result in greater fracture resistance. The risk of fracture is also reduced by the low level of carbide-forming elements, which unfortunately translates into lower wear resistance. X37CrMoV5-1 steel (1.2343) should be considered an intermediate grade between 1.2367 and 1.2714. It contains a moderate molybdenum content.

Microscopic examinations and hardness measurements were carried out on deposited layers produced on tool steels using DO015 filler material, both directly after hardfacing and following post-weld heat treatment. The selection of DO015 was deliberate—prior preliminary tests demonstrated that its application in the hardfacing refurbishment of hot-working tools significantly increases their service life, making it particularly suitable for forging tool regeneration. Post-weld heat treatment was applied to the tested deposited layers, followed by an assessment of its impact on layer quality. The aim of the study was to evaluate the potential for improving the quality of deposited layers on tool steels through heat treatment. To this end, hardening and tempering were applied to improve the quality of the deposited layers and restore the proper microstructure in the heat-affected zone.

### 3.1. Hardfacing Proces

**Figure 4:**
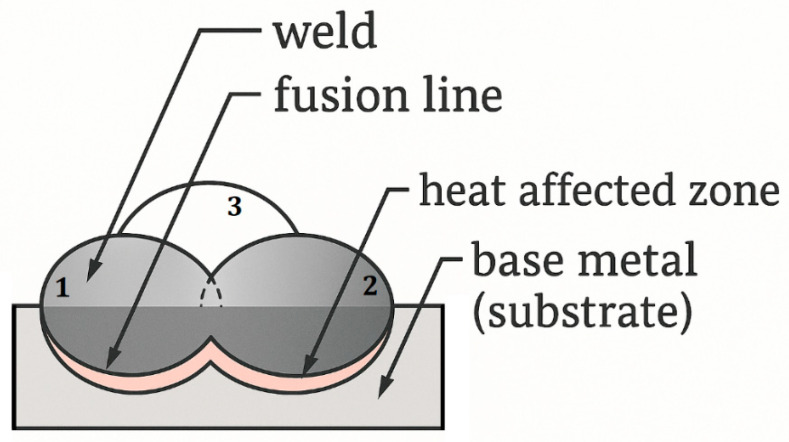
Hardfacing diagram.

The hardfacing process was carried out by means of robotized gas metal arc welding. The GMAW hardfacing was performed by using Lorch SpeedPulse S power source (Lorch Schweißtechnik GmbH, Auenwald, Germany) and TOUGH GUN ThruArm robotic gun. The torch was mounted and manipulated by a six-axis robot Yaskawa Motoman HP20 (Yaskawa, Japan). Endotec DO*15 cored wire with a diameter of 1.2 mm was used as a filler material. This is an iron-based feedstock wire with low chromium content. The deposit is characterized by high resistance to cracking and long-term impact, and can also be used at elevated temperatures, up to 600 °C. The mixture of Ar + 2.5%CO_2_ was used as a shielding gas here. The hardfacing parameters are collected in Table 2 [40], whereas the schematic diagram illustrating the deposition scheme of a three-pass (1, 2, 3) hardfacing deposit is presented in Figure 4. Each substrate material was in the form of a cube with the dimensions of 100 × 100 × 50 mm^3^.

The chemical composition of the hardfaced material is presented in Table 3. Studies conducted as part of this work [40] indicate that chemical homogeneity is achieved only in the third deposited layer. For this reason, the chemical composition was determined based on three-layer hardfacing applied to all three tested steels, and the results are presented as the average values from these deposited layers. In terms of chemical composition, the hardfacing material was most similar to X37CrMoV5-1 steel, although it showed higher contents of carbon and manganese, as well as elevated levels of alloying elements. It also contains tungsten, which is absent from the tested tool steels.

### 3.2. Post-Weld Heat Treatment (PWHT)

**Figure 5:**
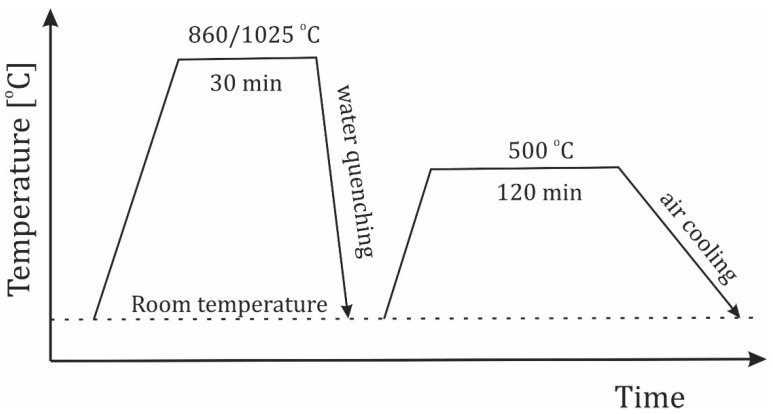
Heat treatment scheme.

Heat treatment was performed on the deposited layers. The heat treatment parameters were selected based on standard recommendations for hardening and tempering of individual steel grades. The hardening and tempering temperatures and holding times are listed in Table 4. Heat treatment schemes are shown in Figure 5.

## 4. Experimental

Metallographic studies were performed on reference specimens consisting of two-layer hardfaced deposits, both before and after heat treatment. The observed microstructural changes accurately reflect those occurring in refurbished tools.

### 4.1. Examination of Modified X38CrMoV5-3 Steel

**Figure 6:**
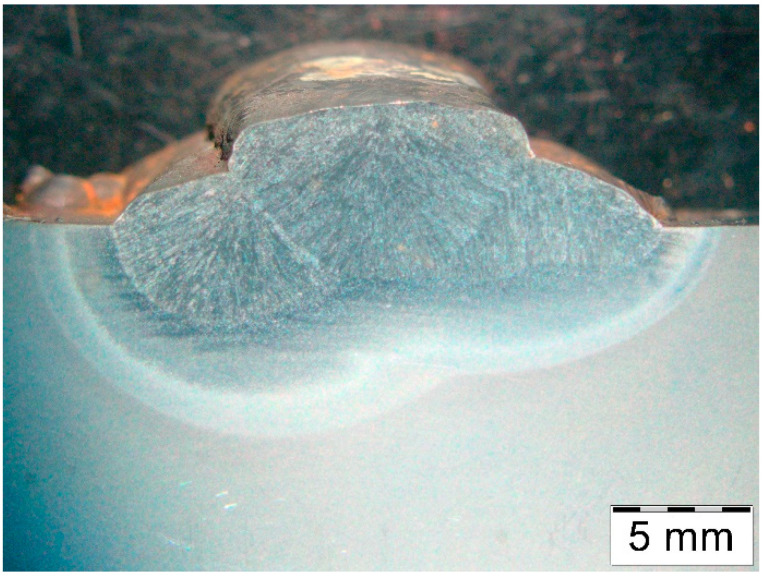
Macrostructure of the deposited layer produced on modified X38CrMoV5-3 steel.

The macrostructure of the deposit deposited on modified X38CrMoV5-3 steel is shown in Figure 6.

**Figure 7:**
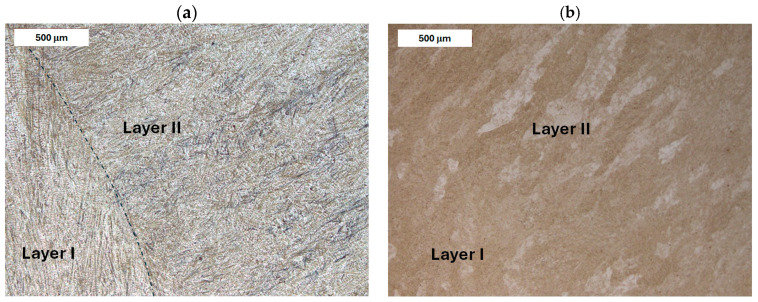
Microstructure of the deposit obtained on modified X38CrMoV5-3 steel. Transition zone between two overlapping weld beads of the deposit: (**a**) before PWHT and (**b**) after PWHT. Light microscopy, etched condition.

**Figure 8:**
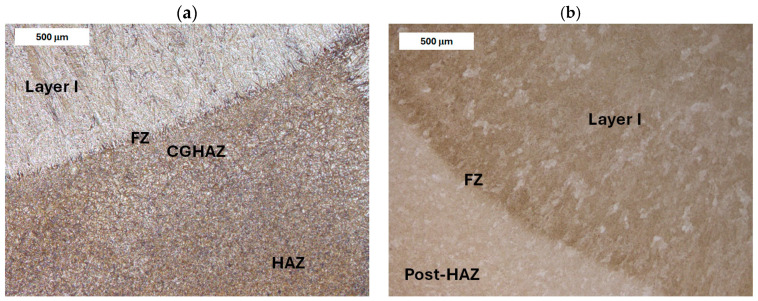
Microstructure of the deposit produced on modified X38CrMoV5-3 steel. Fusion zone between the deposited layer and the HAZ: (**a**) before PWHT and (**b**) after PWHT. Light microscopy, etched condition.

**Figure 9:**
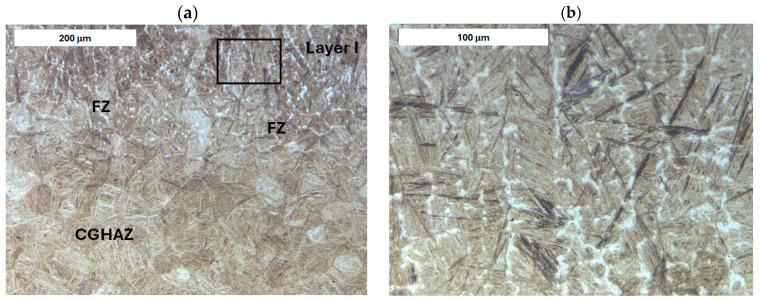
Microstructure of the deposit on modified X38CrMoV5-3 steel before PWHT: (**a**) fusion zone between the deposit and the CGHAZ and (**b**) magnified area from image (**a**). Light microscopy, etched condition.

**Figure 10:**
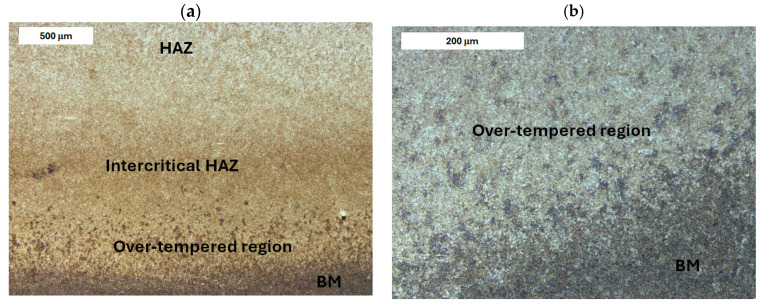
Microstructure of the deposit on modified X38CrMoV5-3 steel before PWHT: (**a**) HAZ adjacent to the base material (BM) and (**b**) magnified area from image (**a**). Light microscopy, etched condition.

The microstructure of the deposited layer produced on modified X38CrMoV5-3 steel is shown in Figure 7, Figure 8, Figure 9 and Figure 10. The microstructure of the deposited layer consisted of hardening structures such as martensite and bainite (Figure 7a). The microstructure of the deposit exhibited a cellular-dendritic morphology with a considerable amount of retained austenite, observed mainly in the interdendritic regions. The bainite content varied between the individual layers of the hardfaced deposit. In the areas of the deposit distant from the base material (BM), the structure was predominantly martensitic, with individual needle-like bainite formations visible mainly at higher magnifications. After heat treatment, the structure of the deposited layer became clearly more homogeneous. Only tempered martensite was observed within the deposit area (Figure 7b).

Before heat treatment, significant structural changes were observed in the heat-affected zone (HAZ). Directly adjacent to the deposited layer was the *Coarse Grain Heat-Affected Zone* (CGHAZ)—Figure 8a. This zone lies immediately beyond the *Fusion zone* (FZ), where temperatures approach just below the melting point of the base material. Such high temperatures cause austenitization and the dissolution of carbide precipitates, which leads to increased grain boundary mobility and rapid grain growth. An enlarged view of the FZ and CGHAZ is shown in Figure 9. Further from the FZ was the *Intercritical HAZ*. This zone is associated with temperatures between the lower transformation temperature (A_C1_) and the upper transformation temperature (A_C3_), constituting a region of partial phase transformation [41]. Closest to the unaffected material was the *Over-tempered* (OT) region. In this area of the HAZ, tempering-related structural changes were observed (Figure 10). After heat treatment, the HAZ showed a clearly more uniform structure. Grain refinement was also observed in the CGHAZ area (Figure 8b).

**Figure 11:**
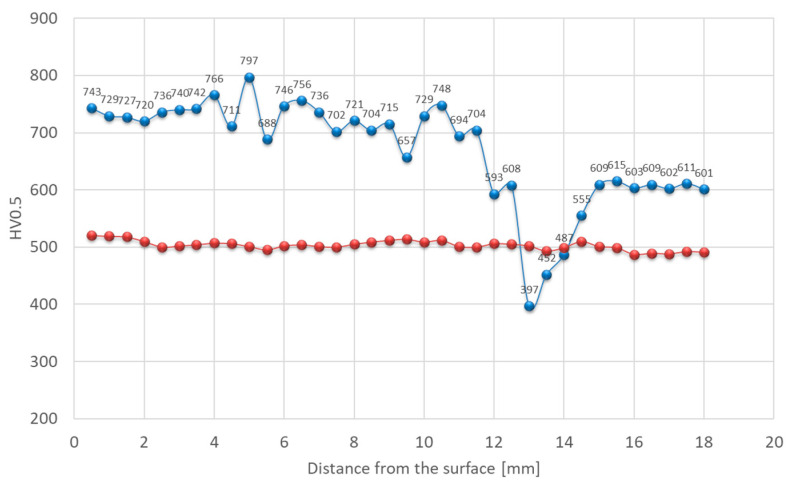
Hardness distribution across the deposit on modified X38CrMoV5-3 steel before PWHT (blue line) and after PWHT (red line).

Hardness was also affected. In the as-welded state, there is a significant variation in the hardness of each zone of the welded joint. Hardness exceeding 700 HV0.5 was observed within the deposit area, while the base material reached approximately 610 HV0.5 (Figure 11). Structural changes in the HAZ were accompanied by a decrease in hardness. This is associated with the tempering that occurred in this area, exceeding the secondary hardness peak temperature observed on tempering curves of quenched steels as a function of hardness. As a result, a softened zone was observed in this area, where hardness dropped to approximately 400 HV0.5. These maximum and minimum values represent the amounts of hardening and softening of 11% and 33%, respectively, compared with the BM. For deposited layers subjected to heat treatment, a stable hardness gradient was achieved. Chemical composition plays a secondary role in strengthening this material [40].

### 4.2. Examination of X37CrMoV5-1 Steel

**Figure 12:**
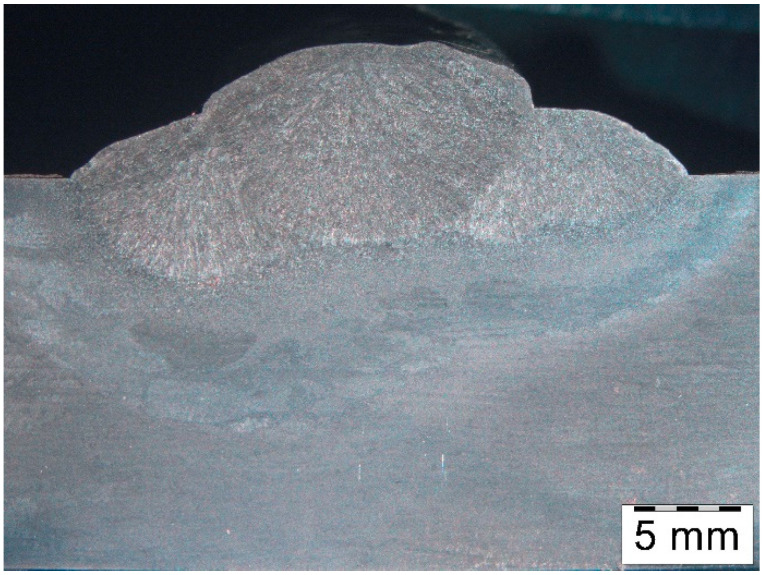
Macrostructure of the deposit obtained on X37CrMoV5-1 steel.

Macrostructure of the deposit on X37CrMoV5-1 steel is shown in Figure 12.

**Figure 13:**
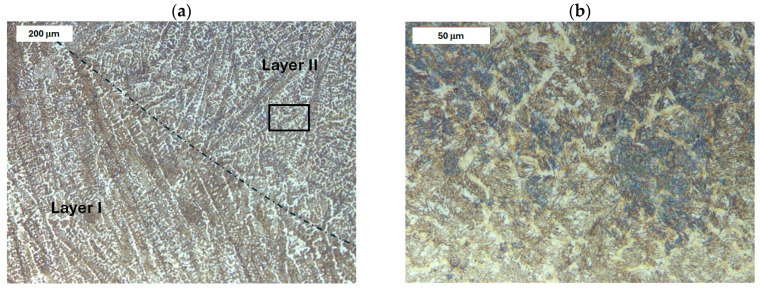
Microstructure of the deposit on X37CrMoV5-1 steel before PWHT: (**a**) transition zone between the beads and (**b**) magnified area from image (**a**). Light microscopy, etched condition.

**Figure 14:**
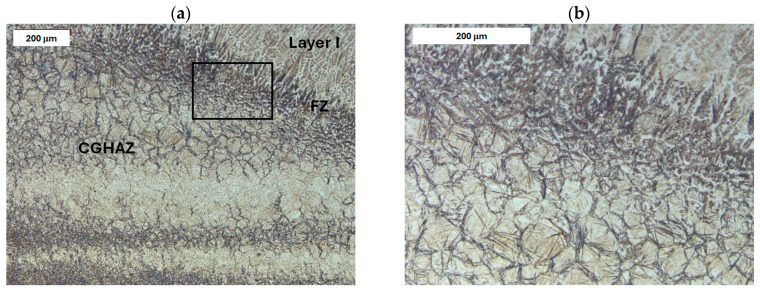
Microstructure of the deposit on X37CrMoV5-1 steel: (**a**) fusion zone between the deposit and CGHAZ before PWHT and (**b**) magnified area from image (**a**). Light microscopy, etched condition.

**Figure 15:**
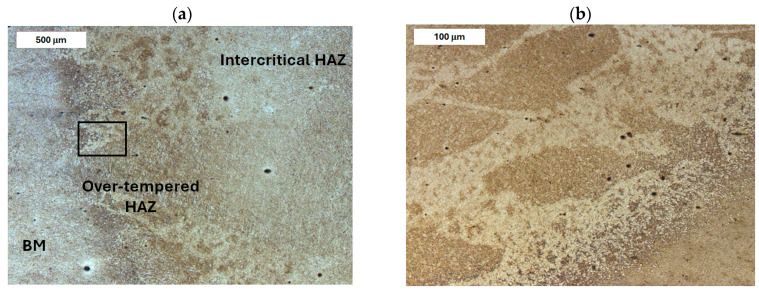
Microstructure of the deposit on X37CrMoV5-1 steel before PWHT: (**a**) HAZ adjacent to the unaffected zone and (**b**) magnified area from image (**a**). Light microscopy, etched condition.

**Figure 16:**
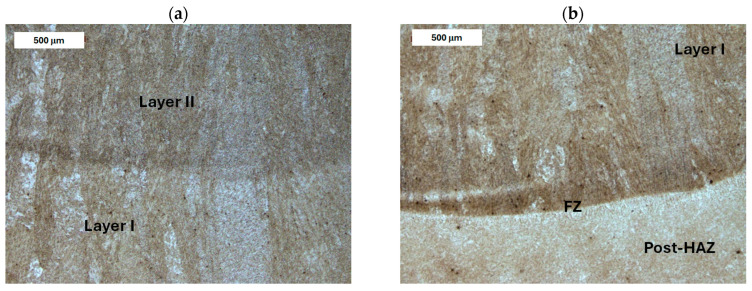
Microstructure of the deposit on X37CrMoV5-1 steel after PWHT: (**a**) transition zone between the weld beads and (**b**) fusion zone between the deposit and HAZ. Light microscopy, etched condition.

In the case of X37CrMoV5-1 steel, the microstructure of the deposit area was similar to that observed in modified X38CrMoV5-3 steel (Figure 13, Figure 14, Figure 15 and Figure 16). It consisted of hardened structures with retained austenite in the interdendritic regions. In the HAZ, grain growth was observed near the deposited layer, accompanied by the formation of a bainitic–martensitic structure. However, in this case, a significantly higher bainite content was observed in the CGHAZ adjacent to the fusion zone than in modified X38CrMoV5-3 steel (Figure 14). Beyond the normalized grain size zone, an over-tempered region was observed, showing a high degree of heterogeneity due to the irregular decomposition of tempered sorbite and the resulting microstructural spheroidization (Figure 15). A varied degree of carbide coarsening was observed, resulting from the tempering stage. After PWHT, as in the previous case, a clearly more homogeneous microstructure was observed. The microstructure of both the deposited material and the base material featured tempered martensite (Figure 16).

**Figure 17:**
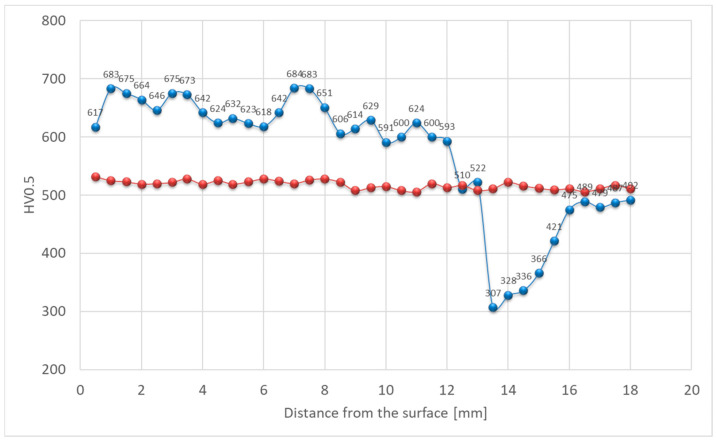
Hardness distribution across the deposit on X37CrMoV5-1 steel before PWHT (blue line) and after PWHT (red line).

In the as-welded condition, in line with the microstructure, the hardness distribution across the weld was non-uniform. Within the deposited layer, hardness ranged from approximately 600 to 700 HV0.5 (Figure 17). In the softened zone, a substantial drop in hardness was recorded, reaching locally as low as 300 HV0.5, while the base material hardness was close to 500 HV0.5. This corresponds to approximately 40% hardening and 40% softening, respectively, compared with BM. The decrease in hardness within the intercritical HAZ was caused by carbide coarsening during tempering. After the PWHT, the hardness distribution was more uniform over the entire cross section. Only minor fluctuations due to inherent heterogeneity were observed (Figure 17). The hardness of the deposited layer and base material after PWHT slightly exceeded 500 HV0.5.

### 4.3. Examination of 55NiCrMoV7 Steel

**Figure 18:**
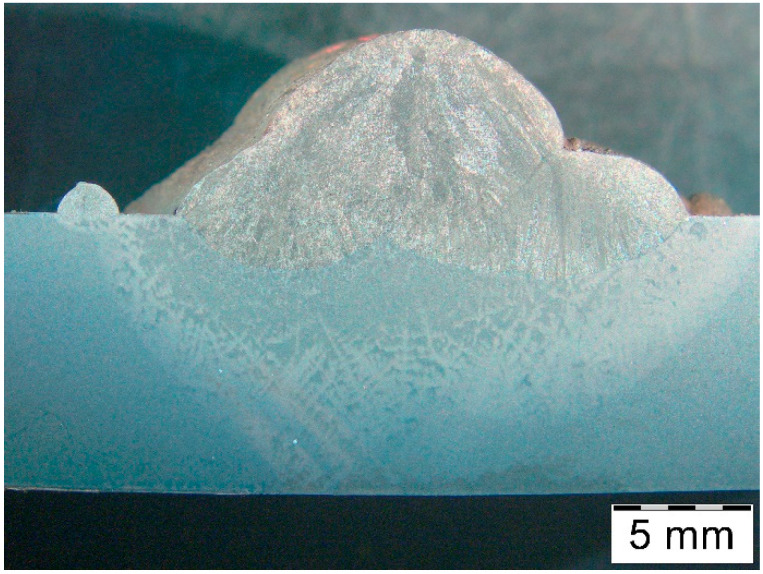
Macrostructure of the deposit obtained on 55NiCrMoV7 steel.

The macrostructure of the deposit on 55NiCrMoV7 steel is shown in Figure 18. At this stage, significant microstructural heterogeneity in the HAZ had already been observed.

**Figure 19:**
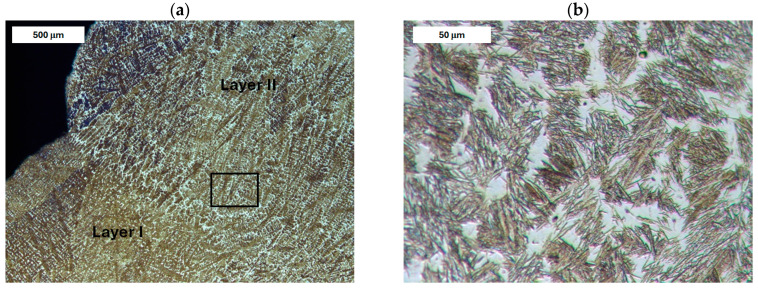
Microstructure of the deposit obtained on 55NiCrMoV7 steel before PWHT: (**a**) transition zone between weld beads and (**b**) magnified area from image (**a**). Light microscopy, etched condition.

**Figure 20:**
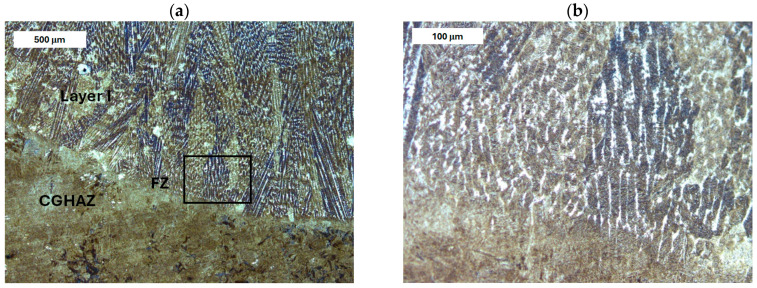
Microstructure of the deposit obtained on 55NiCrMoV7 steel before PWHT: (**a**) fusion zone between the deposit and the HAZ and (**b**) magnified area from image (**a**). Light microscopy, etched condition.

**Figure 21:**
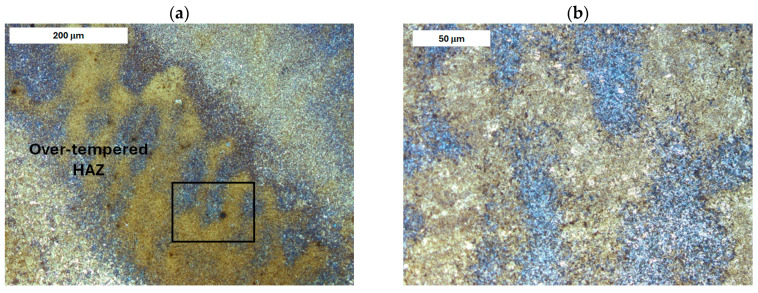
Microstructure of the deposit obtained on 55NiCrMoV7 steel before PWHT: (**a**) HAZ and (**b**) magnified area from image (**a**). Light microscopy, etched condition.

**Figure 22:**
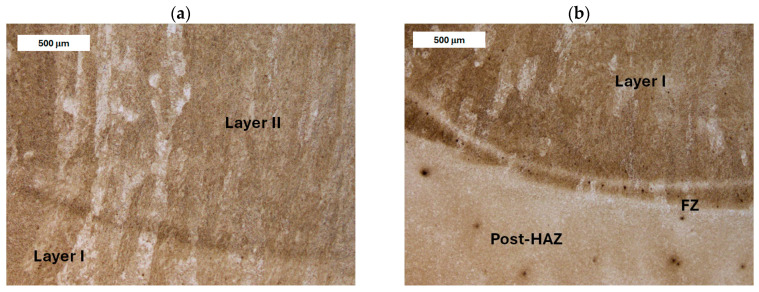
Microstructure of the deposit on 55NiCrMoV7 steel after PWHT: (**a**) deposit area and (**b**) fusion zone. Light microscopy, etched condition.

For this steel, similarly to the previous deposited layers, a martensitic-bainitic microstructure with cellular-dendritic features was observed. A significant amount of retained austenite was present in the interdendritic regions (Figure 19 and Figure 20). The fusion zone showed continuity between the base material and the deposited area (Figure 20). In the HAZ, the microstructure showed considerable variation. Immediately beyond the fusion zone, an overheated area was identified, characterized by coarse-grained martensite and bainite. This zone is expected to have the lowest toughness, which favors crack initiation. With increasing distance from the deposit, the grain size gradually normalized. Further into the HAZ, strong microstructural heterogeneity was observed, with a significant amount of diffusive constituents (Figure 21). In this case as well, PWHT led to the homogenization of the microstructure in both the deposited layer and the base material. Tempered martensite was observed throughout the entire area (Figure 22).

**Figure 23:**
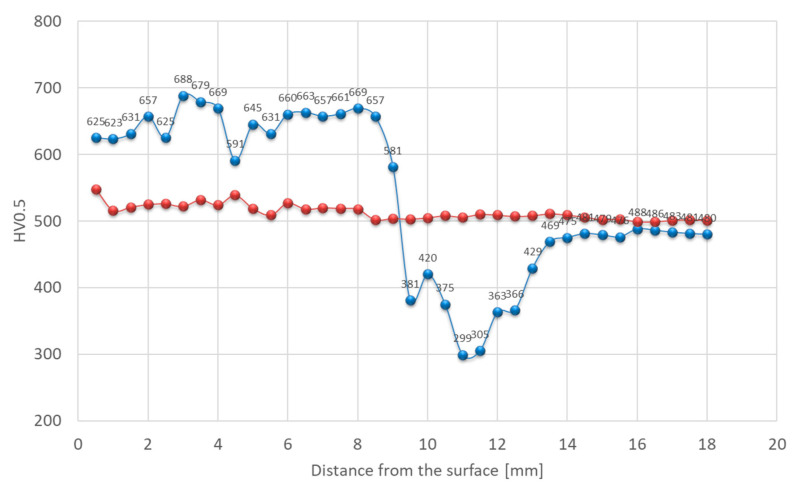
Hardness distribution for the deposit on 55NiCrMoV7 steel before PWHT (blue line) and after PWHT (red line).

Hardness distribution analysis indicates that, in this case, a decrease in hardness was observed in the over-tempered (OT) region, similar in values to that observed for X37CrMoV5-1 steel. Locally, the hardness dropped to approximately 300 HV0.5. This effect was attributed to the relatively low initial hardness of the material, resulting in a higher degree of its strengthening. With the modified X38CrMoV5-3 steel, the hardness in this area was the highest among the tested steels due to the highest content of elements forming hard and stable carbides among the tested steels, as well as the high carbon content. 55NiCrMoV7 steel, despite its higher carbon content, contains the fewest alloying elements. In the weld deposit area made on 55NiCrMoV7 steel, the hardness values ranged from 600 to 700 HV0.5. After post-weld heat treatment (PWHT), the hardness stabilized at around 500 HV0.5 across the entire cross-section of the deposit (Figure 23).

## 5. Discussion

**Figure 24:**
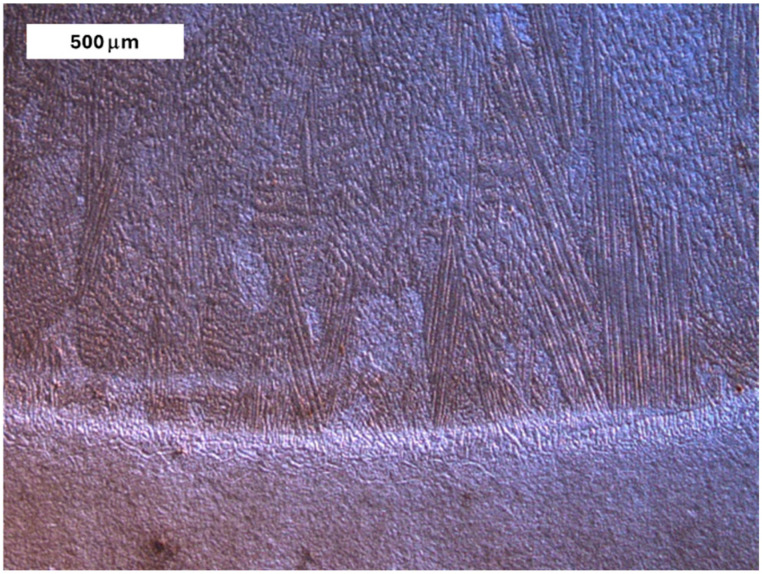
The cast-like structure of the deposit is visible in DIC contrast. Light microscopy, etched condition.

For all tested steels, the initial microstructure consisted of tempered martensite. Due to the thermal cycle of welding, the welded joints developed varied microstructures, depending on the chemical composition of the base material (BM). The thermal gradients during welding promoted local microstructural changes within individual sublayers of the deposited layers. Despite these differences, the affected zones can be categorized into four main regions. Immediately beyond the *fusion zone* (FZ) was the *coarse grain heat-affected zone* (CGHAZ), where grain growth was observed. This was associated with elevated temperatures that enabled austenite grain boundary mobility, resulting in significant grain growth. The large austenite grains led to the formation of coarse hardened structures during cooling. As a consequence of the coarse grain structure, this area is expected to exhibit reduced impact toughness. Farther from the deposited layer, grain size normalization occurred. Next was the *intercritical HAZ*, associated with temperatures between the lower (A_C1_) and upper (A_C3_) critical transformation temperatures, forming a region of partial phase transformation [41]. This area might include a combination of ferrite and pearlite or other phases, depending on the steel grade. Adjacent to the unaffected BM was the *over-tempered* (OT) region. In this area of the HAZ, changes were observed indicating carbide coarsening caused by tempering. The main cause of this phenomenon is the formation of untempered martensite, which is formed by the dissolution of precipitates induced by high cycle heat [42]. Heat treatment did not alter the casting-like nature of the deposited layers, as shown by the microstructure under DIC contrast in Figure 24. A cellular-dendritic character of the microstructure was still observed in the deposit area.

In the as-welded state, significant differences in hardness were observed between the individual zones of the welded joint. These variations are reflected in higher hardness gradients. Very high hardness values were recorded in the deposited layer region before post-weld heat treatment (PWHT). Literature reports indicate that such high hardness levels are typically associated with low impact resistance [38]. The lowest hardness values were noted in the HAZ, specifically in the over-tempered (OT) region. For hot-work tool steels, hardness decreases when the operating temperature exceeds the secondary hardness peak observed on tempering curves. This critical temperature typically falls within the range of 500–540 °C for most hot-work tool steels [43].

Detailed metallographic examinations of deposited layers prior to heat treatment have also been presented in [40]. The presented study confirmed that chemical homogenization of the deposit is achieved after the application of three layers. It was also shown that chemical inhomogeneity does not significantly affect hardness, which is instead primarily determined by the strengthening effect of the martensitic transformation. While differences in chemical composition may lead to minor changes in the microstructure, if the structure is still predominantly martensitic, the observed hardness will be similar. At the same time, above a certain alloying level, further increases do not significantly affect martensitic hardness. These findings are confirmed by the current study. For all tested steels, a uniform hardness of approximately 500 HV0.5 was achieved after PWHT across the entire cross-section of the deposit, despite the evident differences in chemical composition between the base material and the deposited material. Although hardness in the deposited layer was lower than immediately after welding, it still remained relatively high. This reduced hardness in the weld zone should also correspond to improved impact resistance [38]. Before PWHT, the highest hardness, clearly exceeding 700 HV0.5, was observed in the case of the weld deposit made on modified X38CrMoV5-3 steel. As mentioned earlier, this is due to the high content of carbon and alloying elements that form hard carbides. For the other steels, the hardness of the obtained deposits was lower by approximately 50 HV0.5. After PWHT, the material grade and therefore the chemical composition had no significant effect on the final hardness of the deposit. This suggests that at this stage there is no effect related to hardness differentiation due to the precipitation of carbides of different hardness. It cannot be ruled out that using a higher tempering temperature could lead to more pronounced differences in hardness due to the secondary hardening effect. Burja et al. [44] indicate that secondary hardening peaks occur at temperatures higher than 600 °C. Molybdenum, in particular, is an element that strongly influences the effect, while chromium limits secondary hardening and stabilizes M_3_C cementite [45].

## 6. Conclusions

The aim of this study was to assess the potential for improving the quality of deposited layers on three different tool steels through post-weld heat treatment (PWHT). To this end, quenching and tempering operations were applied, which restored the correct microstructure in the heat-affected zone (HAZ). The absence of PWHT resulted in significant microstructural and hardness variations, particularly in the HAZ, where both extremely hard and soft microstructural areas could occur. Such inhomogeneity promotes crack initiation and reduces the resistance of the deposited layer to impact and fatigue loads, which may lead to premature tool failure.

It was demonstrated in this work that post-weld heat treatment (PWHT) significantly improved the microstructural uniformity and hardness distribution of deposited layers on hot-work tool steels. The application of a complete hardening and tempering cycle after hardfacing resulted in a deposited layer with a beneficial, homogeneous microstructure and stable mechanical properties, which should improve durability and crack resistance of the hot forming tools. This observation was valid for all three grades (55NiCrMoV7, X37CrMoV5-1 and X38CrMoV5-3) of hot-work tool steel, despite the differences in chemical composition.

The final properties of the deposited layer are more strongly determined by structural transformations induced by properly selected heat treatment than by the chemical composition of the deposit itself. Therefore, the development of effective welding and heat treatment technologies requires an individual approach tailored to the specific type of tool and tool material in order to ensure the optimal performance of the deposited layer.

Based on the positive results of PWHT presented in this work, the authors plan to extend the research and demonstrate the technology readiness to samples with more diverse shapes, representing the different examples of hot forming tools.

## Figures and Tables

**Table 1:** Chemical compositions of base materials used for hardfacing.

Element, wt.%	C	Mn	Si	S	P	Cr	Mo	Ni	V	Fe
1.2714 (55NiCrMoV7, WNLV)	0.60	0.72	0.23	0.005	0.009	0.97	0.43	1.71	0.08	balance
1.2343 (X37CrMoV5-1, WCL)	0.38	0.33	0.86	0.003	0.011	4.80	1.13	0.10	0.33	balance
Modified 1.2367, (X38CrMoV5-3)	0.50	0.45	0.25	-	0.007	5.10	2.21	0.09	0.50	balance

**Table 2:** Hardfacing parameters.

GMAW Parameter	Hardfacing Current, A	Hardfacing Voltage, V	Gun Speed, m/min	Gas Flow Rate, LPM
Value	260	30	0.3	17–18

**Table 3:** Chemical composition of the deposit obtained in the outermost deposit.

Element, wt.%	C	Mn	Si	Cr	Mo	Ni	V	W	Fe
Hardfacing layer	0.57	0.95	0.74	5.36	1.62	0.06	0.54	1.37	Balance

**Table 4:** Summary of heat treatment parameters.

Grade	Austenising		Tempering	
	Temperature [°C]	Time [min]	Temperature [°C]	Time [min]
1.2714 (55NiCrMoV7, WNLV)	860	30	500	120
1.2343 (X37CrMoV5-1, WCL)	1025	30	500	120
Modified 1.2367, (X38CrMoV5-3)	1025	30	500	120

## Data Availability

The data presented in this study are available on request from the corresponding author. (the data are not publicly available due to privacy).
